# Acellular Extracellular Matrix Scaffolds in Regenerative Medicine: Advances in Decellularization and Clinical Applications

**DOI:** 10.3390/jfb16100383

**Published:** 2025-10-12

**Authors:** Caijun Jin, Xinrui Zhang, Yongxun Jin, Pham Ngoc Chien, Chan Yeong Heo

**Affiliations:** 1 Department of Plastic and Reconstructive Surgery, College of Medicine, Seoul National University, Seoul 03080, Republic of Korea; jcjking96@snu.ac.kr (C.J.); zhangxinrui@snu.ac.kr (X.Z.); jinyongxun789@snu.ac.kr (Y.J.); 2 Department of Plastic and Reconstructive Surgery, Seoul National University Bundang Hospital, Seongnam 13620, Republic of Korea

**Keywords:** decellularization, acellular extracellular matrix, regenerative medicine

## Abstract

Decellularized extracellular matrix (dECM) scaffolds preserve native tissue structure and biochemical cues while minimizing immune responses, creating biomimetic templates that promote cell integration and tissue remodeling. This review examines the current state of dECM research, encompassing decellularization methods, scaffold quality evaluation assays, and tissue-specific applications across dermis, nerve, heart, lung, adipose, and placental ECMs. We analyze commercially available dECM products and ongoing clinical trials, while highlighting recent advances including 3D bioprinting and the integration of dECM with stem cells and growth factors. Despite these promising developments, several challenges continue to limit broader clinical translation: protocol standardization, residual immunogenicity, mechanical durability, and regulatory, manufacturing, and cost barriers. To address these limitations, we outline future directions focusing on patient-specific scaffolds, scalable bioprocessing, and integrated biofabrication strategies that will enable the development of safe and effective dECM-based therapies.

## 1. Introduction

Regenerative medicine represents an evolving interdisciplinary field that seeks to restore or replace damaged tissues by integrating biology, engineering, and clinical science [1,2]. Scaffold-based tissue engineering has become a cornerstone of this field, which employs three-dimensional frameworks to guide cellular organization and facilitate host integration [3,4]. Decellularized extracellular matrix (dECM) scaffolds have emerged as particularly promising within this landscape, as they retain the native tissue’s microarchitecture and biological activity while reducing immunogenic responses—effectively combining the advantages of both natural and synthetic biomaterials [5].

Unlike synthetic polymers, dECM scaffolds naturally retain growth factors, cytokines, and matrix proteins that coordinate cellular adhesion, proliferation, and differentiation [6]. Their tissue-specific biochemical and biomechanical characteristics—including organ-appropriate stiffness and viscoelasticity—make them ideally suited for targeted applications in dermal, cardiovascular, and musculoskeletal repair [7,8]. Through this biomimetic microenvironment, dECM scaffolds facilitate matrix remodeling [9,10], angiogenesis [11,12], and immunomodulation [13,14], establishing their clinical relevance across diverse medical disciplines.

A bibliometric analysis covering 2001–2021 documented a total of 3046 dECM-related publications, including 2700 research articles and 349 reviews, with the United States contributing the largest share [15]. The high-frequency keywords such as immunomodulation, 3D bioprinting and stem cells underscore the diversification and expansion of research topics. Concurrently, the range of approved dECM products has broadened to encompass human dermis, porcine dermis, fetal bovine dermis, porcine bladder, small intestinal submucosa implants, and human peripheral nerve which are employed clinically for skin, cardiovascular, rotator cuff, hernia, and peripheral nerve repairs and breast reconstruction. An increasing number of clinical trials are currently underway to expand the clinical indications for dECM-based therapies, such as, NCT07027735, NCT06606210, NCT06461676, NCT07135596, and so on. Taken together, these trends indicate that dECM research has accelerated and clinical demand is rising, prompting a need for comprehensive evaluations of decellularization techniques and standardization efforts.

**Figure 1:**
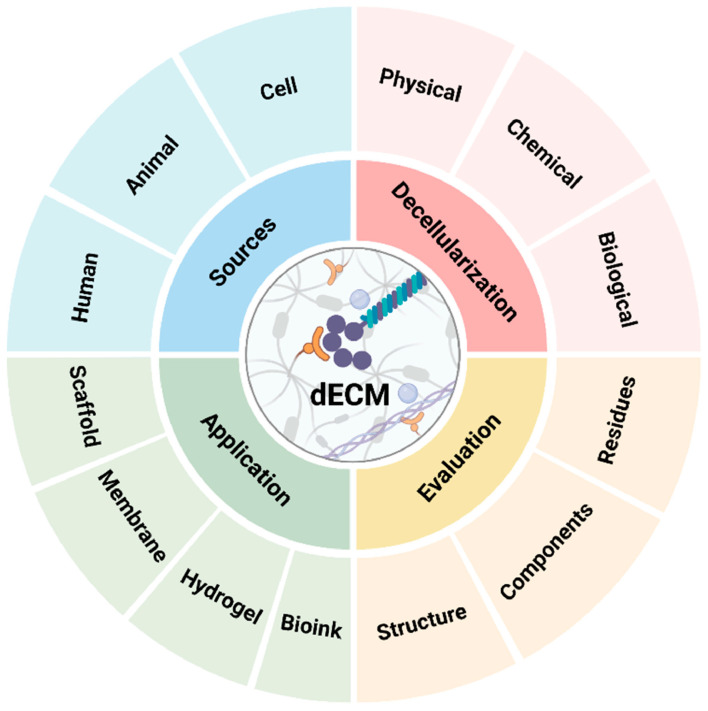
Schematic illustration of the decellularization process, evaluation methods, tissue sources, and biomedical applications of decellularized extracellular matrix (dECM) in skin repair and regeneration. Glycoprotein/laminin-like structure (tan area). Collagen fibrils (blue structure). Proteoglycans and glycosaminoglycans (purple clusters). Elastin or cross-linked ECM fibers (light yellow filaments). Reprinted from Ref. [16].

Building on these advantages, this review provides a comprehensive examination of recent advances in dECM scaffold research (Figure 1). We explore innovations in decellularization methodologies, advanced characterization and evaluation techniques, and the expanding range of clinical applications. Additionally, we discuss critical translational barriers, including standardization challenges, immunogenicity concerns, cost limitations, and regulatory considerations, while proposing future strategies for optimizing scaffold design and accelerating clinical implementation.

## 2. Structure and Function of the Extracellular Matrix

**Figure 2:**
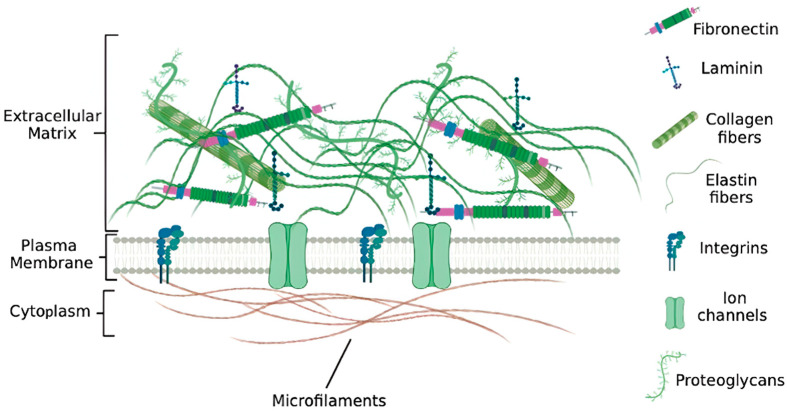
Major extracellular matrix (ECM) components include collagens, laminins, fibronectin, and proteoglycans. These molecules are interconnected by linker proteins such as nidogen and perlecan. Proteoglycans facilitate the organization of collagen fibrils into larger fiber networks, contributing to the structural integrity of the ECM. Reprinted from Ref. [17].

The extracellular matrix (ECM) constitutes a sophisticated three-dimensional supramolecular assembly that confers both biomechanical support and biochemical regulation to resident cells and tissues [17] (Figure 2). As the endogenous biological scaffold that envelops and interconnects cellular populations, the ECM serves critical functions beyond structural maintenance, orchestrating cellular behaviors fundamental to embryonic development, tissue homeostasis, wound healing, and regenerative processes [18]. The ECM comprises a highly organized assemblage of macromolecules, principally categorized into fibrillar proteins, glycosaminoglycans (GAGs), proteoglycans, and matricellular glycoproteins [19]. These constituents demonstrate tissue-specific heterogeneity in composition and stoichiometry, conferring distinctive biomechanical and biochemical properties tailored to organ-specific functional requirement.

Collagens form the primary structural framework of the ECM, representing approximately 30% of total mammalian protein and providing essential tensile strength [19]. Different collagen types serve specialized functions: Type I predominates in skin, tendons, and bone; Type II characterizes cartilage; and Type IV creates the meshwork structure of basement membranes [20]. The hierarchical assembly of collagen—from individual molecules to cross-linked fibrils—maintains tissue biomechanics and structural stability. Working alongside collagen, elastin, and elastic fibers provide resilience and elastic recoil, particularly in mechanically active tissues such as blood vessels, lungs, and skin [21]. This collagen–elastin partnership enables tissues to withstand cyclic mechanical stress while preserving structural integrity. Glycosaminoglycans contribute to ECM function through their unique chemical properties. These linear, negatively charged polysaccharides interact with water and ions to generate osmotic pressure, providing compressive resistance and tissue hydration [22,23]. Key GAGs—including hyaluronan, chondroitin sulfate, heparan sulfate, keratan sulfate, and dermatan sulfate—regulate cellular migration, proliferation, and morphogenesis [24]. Related proteoglycans, which consist of GAG chains attached to core proteins, control matrix hydration, establish permeability barriers, and serve as reservoirs for growth factors and cytokines [25]. These molecules regulate the spatial and temporal availability of morphogens essential for tissue patterning and regeneration [26]. Finally, matricellular glycoproteins such as fibronectin, laminin, vitronectin, and tenascin mediate critical cell–matrix interactions. These proteins facilitate integrin-mediated adhesion, focal adhesion complex formation, and mechanotransduction signaling pathways that translate mechanical forces into cellular responses [27].

The ECM transcends its structural role by actively orchestrating cellular behavior through integrated biochemical signaling and mechanotransduction pathways [28,29]. Cell-ECM interactions, mediated primarily through integrin receptors and syndecans, establish bidirectional communication channels linking the intracellular cytoskeleton with the extracellular microenvironment [30]. These interactions regulate fundamental cellular processes including proliferation, migration, survival, and lineage specification [31]. Integrin-mediated adhesion to ECM ligands initiates signal transduction cascades—including mitogen-activated protein kinase (MAPK), phosphoinositide 3-kinase (PI3K)/Akt, and focal adhesion kinase (FAK) pathways, that modulate gene expression programs and functional phenotypes [32,33]. Mechanical stiffness and topography regulate stem cell fate [34,35], and the ECM stores growth factors such as transforming growth factor-β (TGF-β), vascular endothelial growth factor (VEGF), fibroblast growth factors (FGFs), and bone morphogenetic proteins (BMPs) that are essential for angiogenesis, and immunomodulation [36,37]. During decellularization it is therefore critical to preserve collagen alignment, elastin content, glycosaminoglycans and bound growth factors, because loss of these elements diminishes the scaffold’s capacity to guide cell behavior and tissue regeneration.

Following tissue injury, the extracellular matrix (ECM) undergoes dynamic remodeling that coordinates inflammation, proliferation, and maturation [38,39]. Proteolytic enzymes such as matrix metalloproteinase (MMP) and neutrophil elastase degrade damaged components and release matrikines that modulate immune responses and cell recruitment [40,41]. This transient remodeling facilitates fibroblast and endothelial migration and supports granulation tissue formation. Ultimately, de novo ECM deposition and maturation restore tissue biomechanics and architecture. Importantly, these biological processes highlight why decellularized ECM scaffolds are attractive for regenerative applications. By retaining native structural proteins, GAG, and bioactive cues, dECM can recapitulate the natural wound healing microenvironment, promoting constructive remodeling rather than fibrosis or scar formation.

In conclusion, the ECM functions as a sophisticated biological system that seamlessly integrates structural support with dynamic regulatory mechanisms. The ability to harness this inherent complexity through optimized decellularization and bioengineering strategies presents remarkable opportunities for developing tissue-specific regenerative therapies. Therefore, a thorough understanding of ECM biology is fundamental to designing next-generation biomaterials that can effectively replicate the native regenerative microenvironment and achieve meaningful tissue restoration.

## 3. Decellularization Techniques

**Figure 3:**
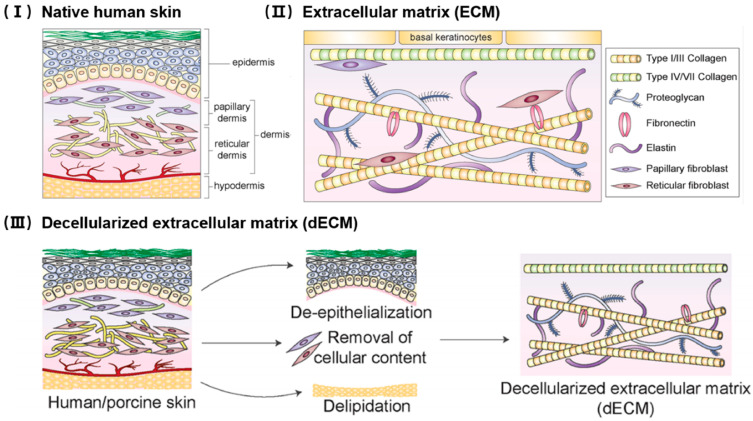
An illustration of the structure of (**I**) natural human skin. Native human skin typically consists of epidermis, dermis, and hypodermis layers. The dermis layer can be subdivided into the upper papillary dermis and the lower reticular dermis, which is resided by the papillary fibroblasts and reticular fibroblasts, respectively. There is vascular plexus in the hypodermis. (**II**) the ECM composition, and (**III**) the dECM production. Reprinted from Ref. [16]. Adapted with permission from Ref. [44]. Copyright 2022 American Chemical Society.

**Figure 4:**
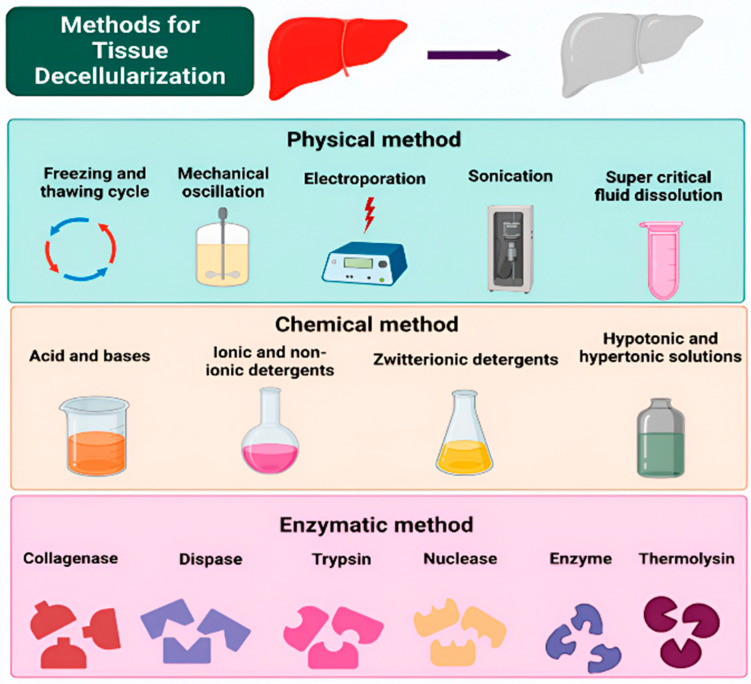
Overview of decellularization strategies used to fabricate acellular extracellular matrix (ECM) scaffolds. In the first row, red indicates native tissue, while gray represents decellularized extracellular matrix. Reprinted from Ref. [57].

Decellularization is a critical bioprocessing technique for creating acellular extracellular matrix (ECM) scaffolds that selectively remove immunogenic cellular components while preserving the native ECM’s structural architecture and bioactive composition [42] (Figure 3). Achieving this delicate balance presents significant technical challenges, as protocols must be tailored to tissue-specific characteristics, including cellular density, vascular architecture, and biomechanical properties [43]. Although substantial methodological advances over the past two decades have enhanced the translational potential of ECM-derived biomaterials, successful outcomes continue to depend heavily on the appropriate selection and combination of decellularization techniques (Figure 4). We summarized and reviewed the current decellularization methods for reference (Table 1).

### 3.1. Chemical Methods

Detergents function as amphiphilic agents that disrupt molecular interactions by solubilizing cell membranes and breaking hydrophobic-hydrophilic bonds [45,46]. These compounds, classified as ionic, non-ionic, or zwitterionic, remove immunogenic material by dissociating lipid membranes and separating DNA from proteins. Ionic detergents such as sodium dodecyl sulfate (SDS), sodium deoxycholate (SDC), and Triton X-100 are particularly effective at disrupting nuclear and cytoplasmic membranes through their targeting of lipid and protein interactions [47,48]. SDS remains widely used in decellularization protocols due to its efficient elimination of cellular and genetic components, though concentration control is critical for optimal results [49]. However, ionic detergents present significant limitations: they can damage ECM proteins and bioactive molecules, while their strong binding to matrix components makes complete removal difficult and may result in cytotoxic effects on subsequently seeded cells. Non-ionic detergents offer a gentler approach that better preserves ECM structure by avoiding protein denaturation [50]. These agents successfully remove membrane lipids and DNA–protein complexes while maintaining collagen orientation and matrix integrity. Despite these advantages, their mild nature limits their ability to completely eliminate nuclear material, often necessitating additional enzymatic or physical treatments to achieve thorough decellularization.

Acids and bases catalyze decellularization through hydrolytic breakdown of cytoplasmic components and nucleic acids. Acidic agents, including peracetic acid (PAA), hydrochloric acid, and acetic acid, disrupt cellular material while providing antimicrobial benefits. PAA demonstrates particular effectiveness by solubilizing organelles, degrading nucleic acids, and sterilizing tissues simultaneously [51]. However, acidic treatments can damage ECM architecture, reduce collagen content, and compromise tissue mechanics [52]. To minimize these adverse effects, low concentrations such as 0.1% PAA are recommended for thin tissues due to their reduced impact on ECM integrity [42,53]. Alkaline solutions, including ammonium hydroxide, sodium hydroxide, and sodium sulfide, promote decellularization by denaturing DNA and inducing cell lysis [54]. High-pH bases above pH 11 prove especially effective at degrading chromosomal DNA [52]. Nevertheless, prolonged exposure to strong bases can compromise ECM structure, decrease GAG content, and eliminate beneficial growth factors. Solutions within the pH 10–12 range may severely damage collagen, fibronectin, and other ECM components, potentially triggering adverse host responses and fibrosis. As with acidic treatments, optimizing both concentration and exposure time is essential to balance effective decellularization with scaffold integrity preservation.

Hypertonic and hypotonic solutions contribute to decellularization by exploiting osmotic pressure to induce cell lysis, dehydration, and death [47]. Hypertonic solutions such as high-concentration saline promote protein removal while preserving basal lamina structures, whereas hypotonic solutions facilitate nuclear and DNA clearance [55]. These treatments offer practical advantages, as they wash out easily and reduce cytotoxic risks from chemical residues. Saline-based treatments using NaCl in PBS have demonstrated minimal impact on ECM transparency, particularly beneficial for sensitive tissues like the cornea. Despite these benefits, osmotic treatments alone cannot achieve complete cell removal. Hypotonic-induced cell lysis may release antigens and cause tissue swelling, limiting their standalone effectiveness. Consequently, these solutions are typically integrated with enzymes or detergents to enhance overall decellularization efficiency [56]. For example, hypertonic-DNase protocols have demonstrated superior DNA removal rates of at least 95% compared to sequential hypertonic-hypotonic washes [55]. While hypertonic and hypotonic solutions provide mild and supportive treatment options, they achieve optimal results when incorporated into comprehensive multi-step protocols.

### 3.2. Enzymatic Methods

Enzymatic approaches provide targeted degradation of specific cellular components, offering enhanced selectivity compared to chemical methods [54]. Endonucleases, including deoxyribonuclease (DNase) and ribonuclease (RNase), catalyze the hydrolysis of phosphodiester bonds in nucleic acids, effectively fragmenting genomic material to minimize immunogenic potential while reducing residual DNA content below clinically acceptable thresholds. Proteolytic enzymes, particularly serine proteases such as trypsin, facilitate cellular detachment through selective cleavage of cell–matrix adhesion proteins.

Trypsin represents one of the most widely employed proteases in decellularization protocols due to its ability to cleave peptide bonds at the carboxyl side of arginine and lysine residues [58]. This enzymatic activity promotes cellular material detachment from the extracellular matrix by breaking down cell adhesion proteins. The enzyme’s effectiveness in removing cellular components without causing cytotoxic effects has made it a standard component in many decellularization protocols [54].

Dispase provides a more specialized approach as a neutral protease that specifically targets fibronectin, collagen IV, and other basement membrane components [59]. This selectivity makes it particularly valuable for decellularizing delicate tissues without extensive ECM ultrastructure damage. Unlike more aggressive proteases, dispase exhibits controlled enzymatic activity that enables effective epithelial cell detachment from underlying matrix layers while preserving basement membrane integrity and the interstitial collagen network. Its application proves especially beneficial for epithelial-rich tissues such as skin, respiratory tract, and glandular organs [60]. Dispase is frequently combined with other enzymes like trypsin and DNase, or mild detergents, to enhance overall decellularization efficiency while minimizing ECM disruption [61,62].

Lipase contributes to decellularization by catalyzing the breakdown of lipid molecules, particularly triglycerides, into glycerol and free fatty acids [63]. This enzyme plays a complementary role by targeting lipid-rich cellular membranes and facilitating cytoplasmic debris removal, proving especially valuable in adipose tissue and glandular organs where lipid accumulation can hinder complete cellular clearance. By disrupting lipid bilayers, lipase enhances the access of other decellularization agents—such as proteases and nucleases—to intracellular targets. The enzyme is commonly used alongside DNase and detergents to improve membrane solubilization and overall decellularization efficiency [64], with successful applications reported in adipose tissue [65] and amniotic membrane protocols [66,67]. Its specificity and relatively low cytotoxicity make it a valuable component of multi-step decellularization strategies focused on preserving ECM structure while ensuring thorough cell removal.

Similarly, phospholipase functions as a lipid-degrading enzyme that hydrolyzes phospholipids in cell membranes, supporting the removal of membrane-bound components during decellularization [68,69]. This enzyme demonstrates particular effectiveness in lipid-rich tissues such as the brain, liver, and adipose tissue, where dense membrane structures can impede cell clearance. When used in combination with agents like trypsin, DNase, or detergents, phospholipase enhances intracellular content removal while maintaining ECM preservation.

### 3.3. Physical Methods

Physical disruption methods serve as essential complements to chemical and enzymatic approaches, enhancing reagent penetration and facilitating cellular debris removal [70]. Freeze–thaw cycling represents a fundamental technique that induces mechanical cell lysis through intracellular ice crystal formation and subsequent membrane rupture [71,72]. While effective for thin tissues and monolayer constructs, this approach shows diminished efficacy in thick, dense tissues where deeper reagent penetration is required. Advanced physical methods have expanded these capabilities through controlled mechanical agitation [73], hydrostatic pressure application [74,75], and vacuum-assisted perfusion systems [76], all of which enhance convective transport of decellularizing agents throughout tissue architecture. However, these techniques require careful optimization, as excessive mechanical forces can induce undesirable ECM deformation, fiber misalignment, or loss of mechanical anisotropy. Perfusion-based decellularization has emerged as the gold standard for whole-organ scaffold generation, utilizing the organ’s native vasculature to achieve uniform reagent distribution while preserving critical microvascular networks [77]. This approach has demonstrated success in complex organs, including hearts [78], lungs [79], uterus [80], and kidneys [81], maintaining organ-specific architecture essential for subsequent recellularization and functional restoration.

Recent technological innovations have introduced sophisticated decellularization modalities that minimize harsh chemical exposure while maintaining processing efficiency. Supercritical carbon dioxide (sCO2) technology exploits the unique solvating properties of supercritical fluids to extract lipids and cellular components under mild temperature and pressure conditions [82,83]. This approach offers exceptional ECM preservation with minimal environmental impact while eliminating concerns regarding toxic residue retention. Ultrasound-assisted decellularization utilizes acoustic cavitation phenomena to enhance mass transfer and accelerate cellular removal, with controlled ultrasonic energy creating microstreaming effects that improve reagent penetration without compromising matrix ultrastructure [84]. Similarly, irreversible electroporation (IRE) provides a chemical-free alternative through high-voltage electrical pulses that induce nanoscale membrane permeabilization, proving particularly advantageous for sensitive tissues [85].

Emerging bioreactor technologies incorporating real-time monitoring capabilities enable dynamic protocol adjustment based on effluent composition analysis and scaffold property assessment, representing significant progress toward standardized, quality-controlled processing [86]. The integration of machine learning algorithms for protocol optimization promises to accelerate reproducible decellularization process development by identifying optimal parameter combinations through iterative refinement [87,88]. Additionally, the convergence of decellularization with advanced biofabrication techniques—including three-dimensional bioprinting and microfluidic tissue engineering—expands possibilities for creating hierarchically organized, vascularized tissue constructs that more closely recapitulate native tissue complexity [89,90].

Decellularization has thus evolved from a rudimentary tissue processing technique to a sophisticated bioengineering discipline requiring precise control over multiple interdependent variables [91]. The expanding repertoire of chemical, enzymatic, physical, and hybrid methodologies enables the production of tissue-specific ECM scaffolds that preserve essential regenerative cues while minimizing immunogenic potential [50]. Numerous studies have compared different decellularization strategies with respect to cellular removal, ECM preservation and biocompatibility. In human umbilical cord tissue, the single use of trypsin, Triton X-100 or sodium deoxycholate failed to meet residual DNA criteria within a short treatment time, whereas a sequential combination of trypsin/EDTA, Triton X-100 and sodium deoxycholate achieved the lowest dsDNA content, exhibited minimal cytotoxicity, and retained collagen and elastin at levels comparable with native tissue [92]. Similarly, in large-animal tendon decellularization, protocols incorporating freeze–thaw cycles followed by Triton X-100 incubation reduced residual nuclei and DNA to 1% and 20%, respectively, while maintaining ECM ultrastructure. SDS-based protocols removed DNA efficiently but induced greater matrix alterations and impaired recellularization [93]. Collectively, these comparisons indicate that combining mild detergents with enzymes and physical treatments often yields a better balance between decellularization efficiency and biocompatibility than single-agent approaches.

However, successful decellularization requires careful balance between efficacy and preservation. While enzymatic and chemical agents such as endonucleases, dispase, ionic and non-ionic detergents, acids, and bases effectively remove cellular components, excessive concentrations or prolonged exposure can denature structural proteins, deplete glycosaminoglycans and growth factors, and weaken scaffold mechanical strength [94,95]. Therefore, each protocol must balance decellularization efficiency with preservation of ECM composition, and washing steps should ensure complete removal of cytotoxic residues [96].

Critically, evidence across multiple tissues suggests that multi-step decellularization protocols tailored to tissue thickness and composition provide the best trade-off between efficiency and biocompatibility. Mild, non-ionic detergents paired with enzymatic agents and augmented by physical methods such as freeze–thaw cycles can achieve thorough cellular removal while preserving ECM integrity. When more aggressive ionic detergents like SDS are necessary for dense tissues, they should be used at carefully controlled concentrations and followed by extensive washing to reduce cytotoxic residues. To facilitate clinical translation, future studies should optimize reagent concentrations and exposure times, employ quantitative assays, such as DNA content, collagen/GAG retention, cytotoxicity, and report standardized metrics to enable robust comparisons across protocols.

**Table 1:** Comparison of decellularization techniques.

Category	Sub-Method	Merits	Demerits	Limitations/Notes	References
Chemical	Ionic detergents (SDS, SDC)	Highly effective at DNA/protein removal	Denatures ECM proteins, cytotoxic residues	Requires extensive washing; risk of growth factor loss	[45,46,47,48,49,50]
	Non-ionic detergents (Triton X-100, Tween-20)	Mild, preserves collagen alignment	Less efficient DNA removal	Often combined with enzymes for completeness	[45,46,47,48,49,50]
	Acids/Bases (Peracetic acid, NaOH, NH_4_OH)	Good sterilization, DNA solubilization	Collagen/GAG degradation, mechanical weakening	Best for thin tissues; high pH damages ECM	[51,52,53,54]
	Hyper/Hypotonic solutions (NaCl, DI water)	Simple, inexpensive, low cytotoxicity	Incomplete decellularization	Usually adjunctive, not sufficient alone	[55,56]
Enzymatic	Nucleases (DNase, RNase)	Specific nucleic acid removal	Expensive, incomplete if penetration limited	Needs detergents/physical methods adjunct	[54]
	Proteases (Trypsin, Dispase, Collagenase)	Efficient membrane/cell protein removal	Can degrade ECM proteins if overused	Must optimize time/concentration	[58,59,60,61,62]
	Lipases	Remove lipids in adipose tissue	ECM damage with prolonged exposure	Narrow use cases	[63,64,65,66,67]
	Phospholipase	Degrade membrane phospholipids, enhancing cell lysis	May also attack ECM lipids	Useful for adipose or lipid-rich tissues; often adjunct to detergents	[68,69]
Physical	Freeze–thaw cycles	Simple, disrupts cell membranes	Inefficient DNA removal	Good adjunct to chemical/enzymatic	[71,72]
	Agitation/Stirring	Enhances reagent diffusion	Possible shear damage	Widely used in lab-scale protocols	[73]
	High hydrostatic pressure	Preserves ECM architecture, sterilizing	Requires special equipment	Limited tissue size	[74,75]
	Vascular perfusion (whole organs)	Maintains vascular networks, uniform penetration	Technically demanding, costly	Primarily for solid organs (liver, kidney, lung, heart)	[76,77]
	Supercritical CO_2_ extraction	Good for lipid-rich tissues, sterilization	Limited penetration in dense tissues	Still experimental, requires optimization	[82,83]
	Sonication	Assists detergent penetration	Local ECM disruption, heat damage	Best used in small samples	[84]
	Irreversible electroporation (IRE)	Creates nanopores in cell membranes without harsh chemicals; preserves ECM	Requires precise control; risk of local ECM disruption	Promising for thick tissues, still experimental	[85]
	Bioreactor systems (dynamic decellularization)	Provides controlled flow, shear, and nutrient removal; scalable	Requires high cost infrastructure	Essential for whole organ engineering	[86]
	Biofabrication (3D printing + dECM bioinks)	Enables patient-specific scaffolds, integration with cells/growth factors	Requires standardization, mechanical strength challenges	Bridges decellularization with regenerative manufacturing	[89,90]

## 4. Characterization of Decellularized ECM Scaffolds

After decellularization, evaluations for dECM must be carefully evaluated to confirm that cellular components are fully removed without damaging the extracellular matrix (ECM). Insufficient decellularization can trigger immune responses, while harsh processing may compromise the ECM’s structure and function. To ensure quality, decellularization protocols require standardized, multi-angle assessment (Table 2).

### 4.1. Evaluations for Cellular Residues

Completely eliminating nuclear and cytoplasmic material from decellularized ECM is difficult, and leftover cell residues can cause inflammation at the implantation site, potentially leading to repair failure [97]. For this reason, it is essential to assess residual cellular content before use. While a standardized protocol is not yet established, both qualitative and quantitative methods are available to evaluate these residues [98].

The most common quantitative measure of successful decellularization is the amount of residual double-stranded DNA (dsDNA). A well-processed ECM should contain less than 50 ng of dsDNA per mg of dry weight, with DNA fragments shorter than 200 base pairs [99]. Residual DNA can be measured using PicoGreen assays [100], spectrophotometry [101], or gel electrophoresis. Shorter DNA fragments are less likely to trigger immune responses, making their reduction an important marker of decellularization quality.

The qualitative evaluation of cellular residues also is measured by Hematoxylin eosin (H&E) staining that is widely used to assess tissue structure and detect cell nuclei [102,103]. The lack of basophilic nuclear staining suggests successful removal of cellular content. For more sensitive detection, fluorescent dyes such as DAPI and Hoechst can be used to visualize residual nuclear material under a fluorescence microscope [104,105]. These histological methods also aid in evaluating ECM preservation, such as collagen fiber alignment and the continuity of the basement membrane, which are important for maintaining the scaffold’s structural and functional integrity [106]. Moreover, immunofluorescence staining can evaluate the intracellular protein like MHC I and II, actin or histones to confirm the removal of immunogenic cell remnants [107]. At the same time, identifying ECM markers such as collagen I, fibronectin, or laminin helps assess the preservation and structural integrity of the scaffold.

### 4.2. Evaluations for dECM Components

Assessing the preservation and integrity of ECM components is a key aspect of evaluating decellularized ECM scaffolds [108]. These components including GAGs, structural proteins, such as collagen, elastin, laminin and fibronectin, and signaling molecules, such as TGF-β1, VEGF and bFGF are crucial for maintaining the scaffold’s biological function and mechanical stability. Loss or degradation of these molecules during decellularization can compromise cell–matrix interactions and reduce the regenerative potential of the scaffold. Many commercial assay kits such as FASTIN (Biocolor Ltd., Carrickfergus, UK), SIRCOL (Biocolor Ltd., Carrickfergus, UK), and BLYSCAN (Biocolor Ltd., Carrickfergus, UK) can be used to quantify key components of elastin, collagen, and GAGs.

### 4.3. Evaluations for Cytocompatibility and Immunogenicity

Before using decellularized scaffolds in vivo, it is important to evaluate their biocompatibility and potential cytotoxicity. One key step is to measure any residual chemicals left from the decellularization and sterilization processes, as these agents especially detergents like SDS can be harmful to cells and interfere with recellularization [49,109]. Studies, such as by Zvarova et al., have identified threshold levels for SDS cytotoxicity with methoylene blue-based colorimetric assay [110].

The most common approach to assess biocompatibility is through in vitro cell culture tests, which can be performed using indirect or direct contact methods [111]. In indirect assays, cells are exposed to media extracts from the scaffold, and their viability and metabolic activity are measured using assays like Live/Dead staining [112], DNA quantification, or MTT. In direct assays, cells are seeded directly onto the scaffold to observe their attachment, morphology, and proliferation [113]. These evaluations are essential to confirm that the scaffold is safe and supports cell growth before clinical application.

**Table 2:** Common assays for evaluating decellularized ECM (dECM) quality.

Assay/Method	Target	Detection Limit/Sensitivity	Advantages	Limitations/Notes	References
DNA quantification (PicoGreen, qPCR)	Residual nuclear material	~50 ng/mg tissue	Highly sensitive, quantitative	Cannot distinguish intact vs. fragmented DNA; requires extraction	[99,100,101]
Histology (H&E, DAPI, Hoechst)	Cellular remnants, nuclei	Semi-quantitative	Simple, visual localization of residual cells	Limited sensitivity, observer bias	[102,103,104,105,106]
Immunohistochemistry/IF	ECM proteins (collagen I/III, laminin, fibronectin) or signaling molecular (TGF-β1, VEGF and bFGF)	N/A	Protein-specific, spatial localization	Antibody-dependent, qualitative	[107]
Biochemical assays (FASTIN, SIRCOL, BLYSCAN)	Elastin (FASTIN), collagen (SIRCOL), GAGs (Blyscan)	1–5 μg/mL	Quantitative, widely used	Destructive, requires standard curves	[108]
Residual detergent assays (methylene blue for SDS)	Chemical residues	μg/mL	Safety-relevant, quantitative	Assay-specific, not always standardized	[110]
In vitro cytocompatibility (MTT, CCK-8, Live/Dead, cell adhesion assays)	Cell viability and proliferation	N/A	Functional, directly relevant	Cell-type dependent; semi-quantitative	[111,112,113]

## 5. Tissue-Specific ECM Scaffolds

The source tissue selected for acellular matrix (ACM) fabrication plays a key role in determining the scaffold’s structure, biochemical profile, mechanical properties, and suitability for specific clinical applications [54]. Different tissues have been decellularized for regenerative medicine, each providing unique advantages tailored to the needs of the target tissue or organ [114]. For example, dermis-derived matrices, rich in collagen and elastin, are widely used for skin and soft-tissue repair [115]. Nerve-derived matrices preserve aligned collagen and laminin, making them suitable for guiding axonal growth in peripheral nerve injuries [116]. Cardiac dECM retains myocardial cues and is being explored in patch or hydrogel form for ischemic heart disease [117]. Other sources, such as lung, liver, adipose, and placental tissues, provide organ-specific environments that are being investigated for corresponding regenerative applications. The following is a summary of commonly used tissue sources for ACM, highlighting their key features and relevance in clinical use.

### 5.1. Skin and Dermis

The dermis is one of the most extensively utilized tissues for producing acellular matrix scaffolds due to its structural richness, high collagen content, and favorable mechanical properties [118]. It provides a dense, fibrous extracellular matrix (ECM) primarily composed of collagen types I and III, along with elastin, fibronectin, and glycosaminoglycans (GAGs), all of which are essential for maintaining tissue integrity and supporting regeneration. These biological components remain largely intact when appropriate decellularization techniques are used, making dermal ECM highly suitable for various clinical applications [119].

Decellularized dermal scaffolds are commonly derived from human or porcine sources. Through controlled physical, chemical, and enzymatic processing, cellular components are removed while preserving the native ECM structure. Decellularized dermal scaffolds are extensively used in various fields such as burn and chronic wound management [120], soft tissue augmentation [121], breast reconstruction [122], hernia and abdominal wall repair [123], dental and periodontal surgery [124]. There are many commercial products include AlloDerm^®^ [125], SureDerm^®^ [126], Strattice^®^ [127], and Integra^®^ [128], each derived from human or porcine dermis and processed with proprietary decellularization protocols.

### 5.2. Peripheral Nerve

The ECM of peripheral nerves consists of distinct layers, including the epineurium, perineurium, and endoneurium, each with specialized structural and biochemical functions [129]. The endoneurial ECM, in particular, is rich in laminin, fibronectin, collagen types I, III, and IV, and various glycoproteins that provide both mechanical support and biological cues critical for neural cell attachment and axonal elongation [130].

Decellularized nerve ECM scaffolds preserve this architecture when processed correctly, maintaining aligned collagen fibrils that support linear axonal growth [131]. The presence of laminin and fibronectin is essential, as they promote neurite outgrowth and direct Schwann cell behavior, while collagen IV forms the basement membrane that stabilizes the regenerated axon-glial interface.

Decellularized nerve grafts are primarily used to bridge nerve gaps in peripheral nerve injuries [132]. Some commercially available or experimentally validated decellularized nerve scaffolds include Avance^®^ Nerve Graft (processed human allograft) and xenogeneic porcine nerve matrices [133]. They serve as guidance channels for axonal regrowth and support the migration and alignment of endogenous or transplanted Schwann cells. Clinical and preclinical studies have shown that dECM nerve scaffolds support containing directed axonal regeneration, myelination by host Schwann cells, functional recovery in sensory and motor nerves [134].

### 5.3. Heart and Vascular Tissue

Cardiac tissues, including the myocardium, pericardium, and heart valves, are increasingly used as sources for decellularized extracellular matrix (ECM) scaffolds in cardiovascular tissue engineering [135,136]. These tissues provide structural complexity, anisotropic mechanical properties, and biologically active matrix components that support cell integration and functional remodeling, making them highly suitable for heart and vascular repair strategies.

Decellularized cardiac tissues have been successfully translated into several clinical products. Bovine pericardium is widely used in cardiovascular surgery and is found in commercial devices such as Edwards Perimount^®^ and CardioCel^®^ which are used in valve replacements, vascular patching, and soft tissue repair [136,137,138]. These scaffolds demonstrate excellent biocompatibility and mechanical performance and are often chosen for their ease of handling and integration with host tissue.

### 5.4. Lung

The lung is a complex organ with highly specialized architecture, making it both a challenging and promising source for decellularized extracellular matrix (ECM) scaffolds in regenerative medicine [139]. Decellularized lung ECM retains critical microstructural and biochemical features essential for supporting respiratory function and provides a native template for lung tissue engineering. With the rising need for alternatives to lung transplantation, decellularized lung scaffolds have gained significant attention for their potential in whole-organ regeneration, disease modeling, and drug testing platforms [140]. Decellularized lung ECM scaffolds have been investigated for a wide range of applications, including whole-lung regeneration, airway reconstruction, disease modeling [141,142].

### 5.5. Adipose Tissue

Adipose tissue has emerged as a promising source for decellularized extracellular matrix (ECM) scaffolds in soft tissue engineering and regenerative medicine [143]. Rich in biologically active molecules and readily available in large quantities through minimally invasive procedures, adipose-derived ECM offers a soft, pliable microenvironment ideal for cell survival, angiogenesis, and tissue remodeling [144]. Decellularized adipose tissue (DAT) scaffolds have shown utility in diverse applications including wound healing, soft tissue augmentation, volumetric muscle loss repair, and adipose tissue regeneration. Decellularized adipose tissue can be used in various application as soft tissue reconstruction, wound healing, adipose tissue engineering and volumetric muscle loss and dermal repair [145,146,147,148].

### 5.6. Placenta

The placenta, including its fetal-derived membranes (amnion and chorion) and maternal tissue, has gained increasing attention as a rich and accessible source of decellularized extracellular matrix (dECM) scaffolds [149,150]. As a temporary organ, the placenta is naturally designed to support tissue growth, immunotolerance, and vascular exchange during fetal development, making its ECM an attractive material for regenerative medicine [151]. Placental-derived dECM scaffolds are particularly valued for their anti-inflammatory, low immunogenic, and angiogenic properties, and have been applied in wound healing, ophthalmology, urology, and soft tissue repair [152,153].

### 5.7. Kidney

Decellularized kidney scaffolds have attracted substantial attention for renal tissue engineering because of their highly specialized microvascular architecture and functional complexity. Whole-organ decellularization of porcine and rodent kidneys has demonstrated that perfusion-based protocols can preserve the native nephronal arrangement, vascular hierarchy, and glomerular basement membrane [154,155]. Such preservation is critical, as renal filtration and tubular reabsorption require not only biochemical cues but also precise three-dimensional alignment. Recellularization studies using endothelial cells, mesenchymal stem cells, and induced pluripotent stem cell (iPSC)-derived renal progenitors have shown partial restoration of perfusion, filtration markers, and limited tubular function [156,157,158,159]. However, challenges remain: achieving uniform cell seeding throughout the dense parenchyma, recreating podocyte–endothelial interactions essential for glomerular filtration, and maintaining long-term perfusion without thrombosis. While clinical translation remains distant, kidney dECM scaffolds provide a critical experimental platform to study renal regeneration and nephrotoxicity, bridging the gap between conventional in vitro models and whole-organ bioengineering.

### 5.8. Liver

The liver has been a new focus of decellularization research due to its clinical demand for transplantation. Perfusion decellularization of porcine [160], rat [161], and human [162] livers has been shown to effectively remove cellular components while preserving the lobular microarchitecture, sinusoidal network, and extracellular matrix proteins. Recellularization efforts with primary hepatocytes, hepatic progenitor cells, and iPSC-derived hepatocyte-like cells have demonstrated partial restoration of albumin secretion, urea synthesis, and cytochrome P450 activity [163,164]. Major hurdles include maintaining long-term hepatocyte functionality, avoiding scaffold thrombosis upon perfusion, and scaling to clinically relevant human-sized livers. Recent advances in bioreactor culture [165], growth factor delivery [166], and hybrid biofabrication strategies [167] aim to address these barriers.

### 5.9. Alternative Sources

While dECM derived from human or animal provide biochemically rich microenvironments, their translational use is constrained by limited donor availability and religious or ethical concerns. These limitations have spurred exploration of plant-derived scaffolds as sustainable alternatives. Decellularized plant tissues offer advantageous physical features, such as large surface area, interconnected networks for fluid transport and pre-existing vascular-like channels, coupled with low cost and minimal ethical barriers [168]. Various species such as apples, spinach, parsley and bamboo have been decellularized; their unique microarchitectures and abundant supply make them attractive for large-scale production [169]. Recent studies also emphasize that plant-based biomaterials are widely accessible, sustainable and provide a broad range of mechanical properties along with innate “vascular” conduits [170,171]. Nevertheless, plant scaffolds lack the complex biochemical cues of mammalian ECM and research remains in its infancy. Standardized protocols, cell–matrix interaction studies and long-term safety assessments are needed before they can complement or replace tissue-derived dECM in clinical applications.

## 6. Clinical Applications and Current Trials

dECM scaffolds have achieved regulatory clearance and are used across a broad range of clinical applications. Commercial products such as AlloDerm^®^ (human dermis) [122], Strattice^®^ (porcine dermis) [172], and Avance^®^ (human peripheral nerve) [133] are applied in reconstructive surgery, including breast reconstruction, hernia repair, and nerve gap bridging (Table 3). Cardiac patches derived from bovine pericardium and SIS-based products like Restore^®^ are widely used in valve and soft tissue repair [136,138]. In ophthalmology, amniotic membrane dECM is utilized for corneal healing [173].

Ongoing clinical trials are expanding applications of dECM to areas such as myocardial infarction [174,175], cartilage regeneration [176,177], chronic wound healing [178,179], and esophageal reconstruction [180]. dECM-based hydrogels are under evaluation for use in endoscopic submucosal dissection [180] and injectable delivery for stroke recovery [181]. Notably, decellularized adipose-derived matrices are under investigation for facial volume restoration and soft tissue augmentation [182]. In orthopedic medicine, cartilage- and meniscus-derived dECM scaffolds are tested in patients with osteoarthritis and traumatic injuries [183]. Meanwhile, placental-derived dECM products are gaining popularity for treating diabetic ulcers and pressure wounds, given their anti-inflammatory and angiogenic properties [184,185,186].

Trials are also evaluating the synergistic use of dECM with stem cell therapies and controlled drug delivery systems [187,188]. Emerging directions include the integration of dECM into bioprinting platforms, customizable patient-specific grafts, and immune-engineered scaffolds for organ repair. Regulatory challenges, variability in source material, and scalability remain key hurdles, but the growing clinical evidence base continues to support dECM as a powerful tool in regenerative medicine.

**Table 3:** Commercial and clinically used ECM/dECM scaffolds.

Product/Brand	Source Tissue	Company/Manufacturer	Indications/Clinical Use	Regulatory/Market Status	Notes	**References**
AlloDerm^®^/AlloDerm RTM/Cymetra^®^ (micronized)	Human dermis	LifeCell/Allergan Aesthetics (AbbVie), Irvine, CA, USA	Breast and abdominal wall reconstruction, chronic wounds, soft-tissue repair	FDA HCT/P; widely used	Gold-standard ADM; micronized Cymetra allows injectable use	[189,190]
DermACELL^®^	Human dermis	LifeNet Health, Virginia Beach, VA, USA	Chronic wounds, breast reconstruction, hernia repair	FDA HCT/P	Matracell^®^ process ensures >97% DNA removal	[191,192]
FlexHD^®^	Human dermis	MTF Biologics, Edison, NJ, USA	Breast reconstruction, hernia repair, soft-tissue reinforcement	FDA HCT/P	Hydrated, pliable ADM	[193,194]
GraftJacket^®^	Human dermis	Wright/Stryker, Kalamazoo, MI, USA	rotator cuff tears, diabetic foot ulcers	FDA cleared	Available in thick or meshed forms	[195,196]
DermaMatrix^®^, AlloPatch^®^ Pliable	Human dermis	MTF Biologics, Edison, NJ, USA	reconstructive surgery	Commercial	Freeze-dried or hydrated formats	[197,198]
SureDerm^®^, CGDerm^®^, MegaDerm^®^/MegaDerm^®^ Plus	Human dermis (Korea)	HansBiomed, CGBio, L&C Bio, Seoul, South Korea	Burn care, plastic reconstruction, dental/periodontal	CE/NMPA/Korean MFDS approvals	Widely used in Asia; Mega Derm Plus recently NMPA-approved (China)	[126]
Strattice™ RTM/ARTIA™ RTM	Porcine dermis (non-crosslinked)	LifeCell/Allergan Aesthetics, Irvine, CA, USA	Abdominal wall/hernia repair, breast reconstruction	FDA 510(k); CE Mark	Multiple versions (Perforated, Extra-thick, Lap)	[199]
XenMatrix™/XenMatrix™ AB	Porcine dermis	BD (C.R. Bard), Franklin Lakes, NJ, USA	Hernia repair, abdominal wall reinforcement	FDA 510(k)	AB version has antibiotic coating (rifampin/minocycline)	[200]
Permacol™	Porcine dermis (crosslinked)	Medtronic, Medtronic Parkway, Minneapolis, MN, USA	Hernia repair, pelvic floor, reconstructive	FDA 510(k)	Long history of clinical use	[201,202]
SurgiMend^®^	Fetal bovine dermis	Integra LifeSciences, Princeton, NJ, USA	Hernia repair, breast reconstruction, soft-tissue reinforcement	FDA 510(k)	Non-crosslinked collagen scaffold	[203,204,205]
Edwards Perimount^®^	Bovine pericardium	Edwards Lifesciences, Irvine, CA, USA	Surgical bioprosthetic heart valves for aortic/mitral valve replacement	FDA and CE approved	One of the most established pericardial valve products with extensive long-term clinical outcome data	[206]
CardioCel^®^	Bovine pericardium	Admedus (now Anteris Technologies), Eagan, MN, USA	Congenital heart defect repair, vascular and pericardial reconstruction	CE Mark; TGA (Australia); FDA clearance for certain indications	Designed to reduce calcification and improve durability; applied in pediatric cardiac surgery	[207]
OASIS^®^ Wound Matrix/Flowable	Porcine small intestinal submucosa (SIS)	Cook Biotech/Smith + Nephew, West Lafayette, TN, USA	Acute and chronic wounds (DFU, VLU, pressure ulcers)	FDA 510(k)	Widely adopted in wound care	[208]
Biodesign^®^ (formerly Surgisis^®^)	Porcine SIS	Cook Biotech, West Lafayette, IN, USA	Hernia repair, fistula plugs, esophageal/gastrointestinal applications	FDA 510(k)	Versatile SIS-based product line	[209]
CorMatrix^®^ ECM	Porcine SIS	CorMatrix Cardiovascular, Roswell, GA, USA	Vascular/arterial and pericardial repair, cardiac applications	FDA 510(k); CE Mark	Used in congenital heart surgery and vascular repair	[210,211]
Cytal^®^, MicroMatrix^®^, Gentrix^®^	Porcine urinary bladder matrix (UBM)	Integra LifeSciences (via ACell), Princeton, NJ, USA	Wound management, hernia repair, pelvic floor	FDA 510(k)	Available in sheets, powders, gels	[212,213]
Endoform^®^/Symphony™/Myriad^®^	Ovine forestomach matrix (OFM)	Aroa Biosurgery, San Diego, CA, USA	Acute and chronic wounds, reconstructive surgery	FDA 510(k); CE Mark	Multiple formats (natural, antimicrobial, injectable, mesh)	[214]
OviTex^®^/OviTex^®^ PRS	OFM reinforced with synthetic polymer fibers	TELA Bio (with Aroa), Malvern, PA, USA	Hernia and abdominal wall reconstruction; PRS version for plastic/reconstructive surgery	FDA 510(k); CE	Reinforced hybrid scaffold	[215]
Avance^®^ Nerve Graft	Human decellularized peripheral nerve	AxoGen, Alachua, FL, USA	Peripheral nerve gap repair	HCT/P in U.S.; transitioning to BLA	Leading nerve ECM product	[216]

## 7. Challenges and Future Directions

Despite the significant progress in dECM development, several challenges limit their broader clinical translation. One major obstacle is the variability in decellularization outcomes due to differences in tissue origin, donor characteristics, and processing protocols [217]. Lack of standardization across laboratories leads to inconsistent scaffold quality and unpredictable host responses.

Another key concern is residual immunogenicity. Incomplete removal of cellular debris, xenoantigens, especially in animal-derived ECM, and decellularization agent residues can trigger inflammatory or immune reactions [50]. Improved protocols that optimize decellularization while preserving ECM functionality are urgently needed.

Mechanical integrity and durability of dECM scaffolds also pose challenges, particularly when applied in load-bearing tissues like heart valves, tendons, and cartilage. Advanced techniques such as mechanical conditioning, crosslinking, or hybrid biomaterials may help enhance biomechanical properties [218,219].

Future directions include personalized ECM scaffolds, derived from patient-specific tissues or designed using 3D bioprinting technologies [220]. Integration with gene editing, stem cells, and controlled-release systems will likely enhance the regenerative potential and specificity of dECM therapies [114]. Additionally, efforts are underway to develop off-the-shelf dECM-derived bioinks, injectable gels, and composite systems for minimally invasive procedures [221]. In terms of characterization, advances in proteomics, transcriptomics, and imaging will also refine scaffold characterization and predict In Vivo performance.

Beyond scientific and technical considerations, successful clinical translation of dECM scaffolds also depends on regulatory approval, scalable manufacturing, and economic viability. Regulatory pathways differ considerably: in the United States, many acellular dermal matrices are regulated under the FDA’s Human Cells, Tissues, and Cellular and Tissue-Based Products (HCT/P) framework, while in Europe most dECM products require CE marking as Class III medical devices, demanding stricter preclinical and clinical evidence. Furthermore, large-scale production must comply with Good Manufacturing Practice (GMP) standards to ensure batch-to-batch consistency, bioburden control, and validated sterilization. These requirements pose logistical and financial challenges, as decellularization relies on donor tissue procurement, rigorous quality testing, and complex supply chains. Finally, economic feasibility is a critical factor: dECM scaffolds are generally more costly to produce than synthetic meshes or alloplastic implants, which can limit adoption in resource-constrained healthcare systems. Cost-effectiveness analyses, incorporating not only material costs but also long-term outcomes such as reduced infection or reoperation rates, will be essential to justify broader clinical implementation of dECM-based therapies. Collectively, overcoming these challenges will pave the way for next-generation dECM scaffolds that are safe, functional, and widely accessible.

The ethical landscape of dECM research encompasses several critical dimensions [222]. First, sourcing and donor consent are fundamental: human-derived dECM is typically obtained from cadaveric donors, living tissue donations, or surgical discards, and must adhere to strict protocols ensuring informed consent, anonymity, and privacy protection. For animal-derived scaffolds, compliance with animal welfare legislation and the 3R principles (Replacement, Reduction, Refinement) is essential to minimize ethical concerns associated with xenogeneic sourcing. Second, xenotransplantation poses unique risks, particularly the potential transmission of zoonotic pathogens. For instance, porcine-derived scaffolds may harbor porcine endogenous retroviruses (PERVs), necessitating stringent donor herd screening, preclinical safety data, and transparent patient consent in line with international xenotransplantation guidelines. Finally, transparency and regulatory oversight are indispensable. Clinical trials should be prospectively registered, with outcomes made publicly available, while regulatory agencies must strike a balance between flexibility to foster innovation and rigor to safeguard patient safety. Together, these considerations underscore that ethical compliance is not peripheral but central to the credibility, acceptance, and sustainable integration of dECM scaffolds into clinical practice.

In summary, the success of dECM scaffolds in regenerative medicine depends on precise characterization of decellularization efficiency, ECM composition, and biocompatibility. Both human and animal sources provide unique advantages across clinical applications. As techniques advance, integrating molecular characterization, mechanical testing, and functional assays will be essential for optimizing next-generation scaffolds.

## 8. Conclusions

Decellularized extracellular matrix (dECM) scaffolds have emerged as a transformative class of biomaterials in regenerative medicine, distinguished by their capacity to recapitulate native tissue architecture and biochemical signaling. The development of effective scaffolds hinges on achieving an optimal balance between thorough cellular material removal and the preservation of the ECM’s structural and functional integrity. This necessitates rigorous quality assessment through standardized methods encompassing DNA quantification, histological staining, and proteomic profiling to ensure scaffold bioactivity and safety.

The versatility of dECM technology is exemplified through tissue-specific scaffolds derived from diverse sources including skin, nerve, cardiac, adipose, lung, and placental tissues. Each scaffold type offers unique regenerative properties specifically tailored to distinct clinical applications, with both human- and animal-derived matrices demonstrating promising efficacy in preclinical and clinical investigations. However, significant challenges persist, particularly the variability inherent in decellularization protocols and the complex regulatory landscape that currently limits widespread clinical adoption.

Contemporary research initiatives are actively addressing these limitations through multiple complementary approaches. These include advancing decellularization methodologies for improved consistency and efficacy, integrating dECM platforms with stem cell technologies and targeted drug delivery systems, and developing innovative scaffold formats such as injectable and three-dimensionally printable constructs. As clinical evidence continues to accumulate and regulatory frameworks mature, dECM scaffolds are positioned to fundamentally transform tissue repair strategies and enable truly personalized regenerative therapeutic approaches.

## Data Availability

No new data were created or analyzed in this study.

## Abbreviations

The following abbreviations are used in this manuscript:

dECM	Decellularized extracellular matrix
GAG	Glycosaminoglycan
ECM	Extracellular matrix
MAPK	Mitogen-activated protein kinase
PI3K	Phosphoinositide 3-kinase
FAK	Focal adhesion kinase
TGF-β	Transforming growth factor-β
VEGF	Vascular endothelial growth factor
FGF	Fibroblast growth factor
BMP	Bone morphogenetic protein
ADAM	A disintegrin and metalloproteinase
MMP	Matrix metalloproteinase
TIMP	Tissue inhibitors of metalloproteinase
DAMP	Damage-associated molecular pattern
PDGF	Platelet-derived growth factor
SDS	Sodium dodecyl sulfate
SDC	Sodium deoxycholate
PAA	Peracetic acid
DNase	Deoxyribonuclease
RNase	Ribonuclease
sCO2	Supercritical carbon dioxide
IRE	Irreversible electroporation
dsDNA	Double-stranded DNA
HE	Hematoxylin eosin
DAPI	4′,6-diamidino-2-phenylindole
MHC	Major histocompatibility complex
ACM	Acellular matrix
GMP	Good manufacturing practice
